# Burn Injury and Explosions: An Australian Perspective

**Published:** 2009-09-16

**Authors:** John E. Greenwood

**Affiliations:** Royal Adelaide Hospital, North Terrace, Adelaide 5000, South Australia

## Abstract

**Objectives**: Increasingly (but not exclusively), terrorist activity and the use of explosive devices have enjoyed the focus of the global media. This paper aims to bring a range of issues to attention, to highlight how burn injuries are sustained in such incidents and why burn injuries (and thus burn disasters) are so complicated to manage. **Materials and Methods**: The author's experience with burn injury caused during explosions and his involvement in burn disaster situations has been summarized to form the basis of the article. This has been expanded upon with discussion points which provide a strategy for planning for such events and by a broad sample of the literature. **Results**: Several strategies are suggested to facilitate planning for burn disasters and to illustrate to those not directly involved why forward planning is pivotal to success when these incidents occur. **Conclusions**: Disasters generating large numbers of burn-injured are relatively frequent. Explosive devices are widespread in their use both in military and increasingly in civilian fields. Encompassing a large range of aetiologies, geographical sites, populations, and resources; burn disaster management is difficult and planning essential.

An incident generating multiple burn injuries is one of the most difficult trauma scenarios to plan for and manage and may be further complicated by the involvement of chemical and/or radioactive agents. Even relatively small numbers of burn casualties have the ability to overwhelm receiving facilities and disasters involving large numbers of burned patients are characterized by appalling mortality.^1^–^4^ There have been more than 240 such incidents in the last 2 centuries and examination reveals most have occurred in the developed world and, although terrorism is an increasing aetiology, accidental fires at mass gatherings such as sporting venues, nightclubs, cinemas, theatres, modes of transport, shopping malls, hotels, and major industrial complexes (especially mining) make up the majority of cases.^5^ More than 15,000 men have died as a result of fire and explosion since 1850 in the US mining industry alone.^6^

Terrorist activity perpetrated by radical organizations occurs frequently on foreign soils and tends to be targeted against first-world democracies, their businesses, or their people; these include the American Embassy bombings in Kenya and Tanzania in 1998,^7^ in which 291 died and more than 5000 were injured and the Kuta nightclub bombings in Bali in 2002, in which 202 died and the exact number of the injured is still unknown.^8^,^9^

Australia has been called the “Lucky Country.”^10^ However, the atrocities of September 11, 2001, and the subsequent involvement of Australian troops in the “War on Terror” in both Afghanistan and Iraq have made Australia and its citizens “legitimate targets” in the eyes of fundamentalists. The attack on Australian holidaymakers in Bali on October 12, 2002, resulted in 88 Australians dying at the scene.

Major bush fires frequently plague New South Wales, Victoria, the Australian Capital Territory, South and Western Australia. Thus far, bushfires have claimed the lives of 640 Australians (including 52 firefighters). Until recently, the worst-single incident was the 1983 Ash Wednesday fires, which devastated Victoria and South Australia, killing 75 (including 16 firefighters).^11^ February 2009 succeeded to this dubious honor, when 400 separate fires in Victoria killed 173 and injured 500 in what has become known as Black Saturday.

## PATTERNS OF BURN INJURY WITH DIFFERING EXPLOSIVE AGENTS

The likelihood of a blast victim sustaining burn injuries, and the type and severity of those injuries, depends largely on the nature of the explosive agent employed. Additional factors related to the victim (such as clothing) and the environment may facilitate burn injuries that are not related to the initial blast per se, but caused by fires generated by the blast.

**Figure 1:**
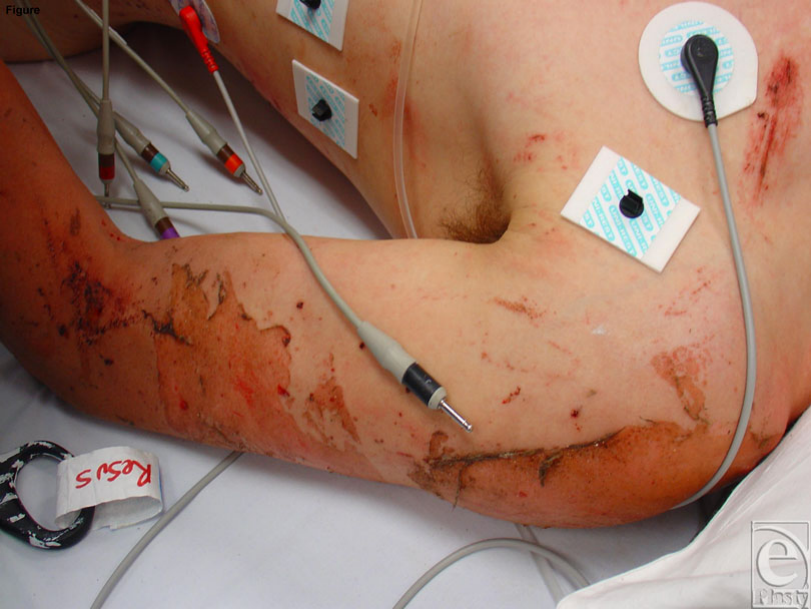
Superficial partial-thickness burns caused by high-explosive detonation during the Gladstone munitions factory explosion in May 2006.

**Figure 2:**
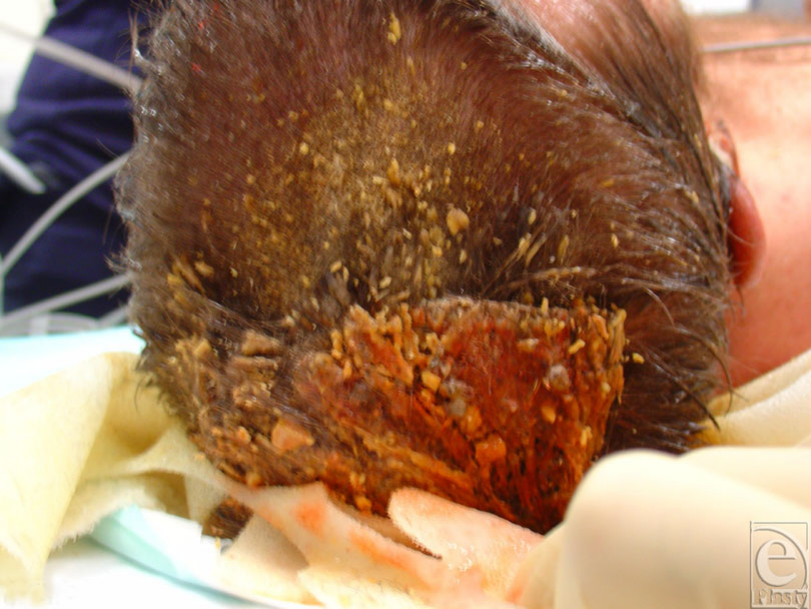
Trinitrotoluene and solidified gum residue firmly adherent to hair and scalp. This residue set to the hardness of concrete and had to be shaved away with a scalpel from the scalp and several other areas of the skin. Whilst this material was in contact with the skin, the patient developed progressively worsening methaemoglobinaemia.^14^

**Figure 3:**
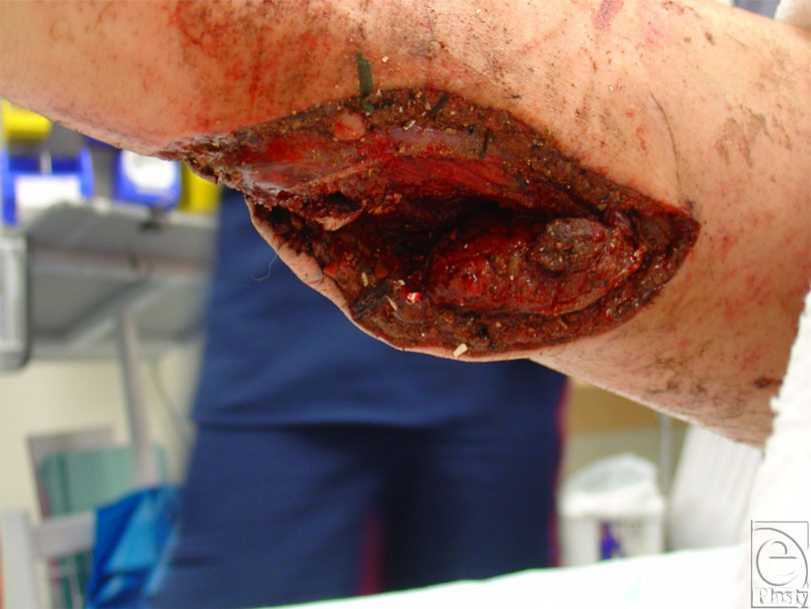
Penetrating calf injury as a result of fragmentation. Note contamination by flecks of the trinitrotoluene/gum residue illustrated in Figure 2.

Blasts caused by conventional high explosives, such as trinitrotoluene, C4, and dynamite, are characterized by the immediate combustion of the explosive with consumption of most of the locally available oxygen.^12^ The instantaneous chemical reaction, while generating enormous pressures, also produces an extremely short-lived, but very high, temperature rise (up to 3000°C).^13^ As a result, burns generated by these types of agents tend to be “flash burns,” which are seldom more than superficial partial thickness and are similar to electrical arc plasma flash injuries. This was perfectly illustrated by the injury patterns demonstrated by the 2 survivors of the Gladstone munitions factory explosion in South Australia (Figs 1–3).^14^

Recent history, in particular the problem of an enemy hiding in tunnels and caves, and the relative impotence of conventional explosives against field fortifications, has led to the development of thermobaric weapons.^15^ These agents, used extensively by the USSR in Afghanistan and more recently by Russia in Chechnya, have been designed to produce heat and pressure effects rather than concentrating on the fragmentation properties of conventional explosives. Instead of most of the explosive energy being expended in breaking the casing and accelerating the casing fragments (employed by conventional high explosives), thermobaric weapons have thin casings enabling most of their energy to be released as blast wave and heat. The resultant fireball and blast wave can travel around corners, penetrating areas inaccessible to conventional bomb fragments, the blast waves being intensified when reflected by walls. Barriers such as sandbags and body armor are not effective against these weapons. Only a fraction of the energy of the explosion is released with the initial detonation. Large volumes of fuel-rich products are generated, which undergo “after-burning” in the postdetonation shock-heated air. This second combustion increases the duration of the blast overpressure and the size of the fireball.^12^ The burn injuries suffered as a result of a thermobaric explosion are much more severe because of the increased magnitude and duration of the fireball.

Other agents capable of producing severe burn injuries are incendiary devices, especially those containing substances such as Napalm. The addition of powdered aluminum soap (or similar) to gelatinize or thicken oil or gasoline increases the viscosity and adhesiveness of the burning material and prolongs the duration of contact and thus the burning insult.^16^

**Figure 4:**
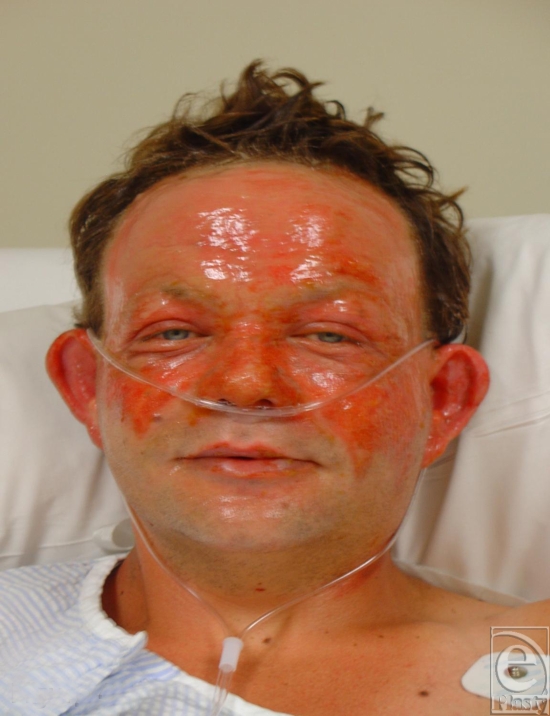
Superficial partial-thickness facial burns caused by the rapid passage of a fireball following a petrol explosion in a confined space.

**Figure 5:**
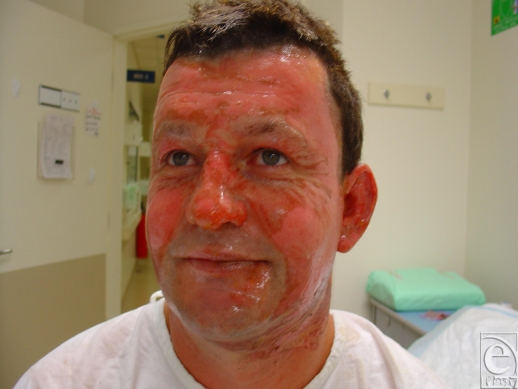
Superficial partial-thickness facial burns from the same petrol explosion displaying the unburned “crow's feet” around the eyes. This gives an indication of the speed and effectiveness of the “blink” reflex in protecting the eyes in these situations.

**Figure 6:**
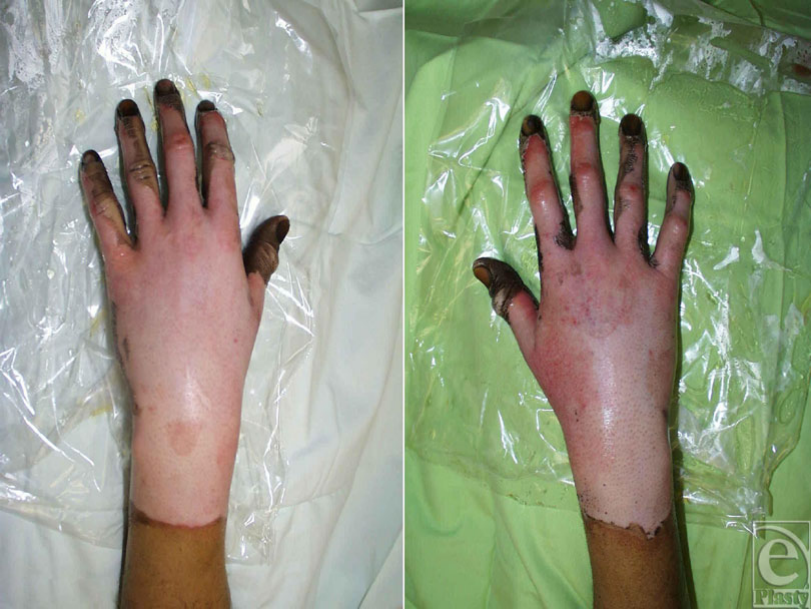
Although exposed areas (Figs 4 and 5) sustain superficial burns, full-thickness burns resulted on these hands as a result of the ignition of clothing. The fireball from the explosion set fire to his gloves.

Improvised explosive devices such as those made from ammonium nitrate fertilizers and “Molotov cocktails” using a liquid accelerant such as petrol are cheap to manufacture^17^ and generate a fireball intermediate to those from conventional high-explosive and thermobaric (volumetric) weapons (Figs 4–6).

Nuclear detonations produce enormous temperature rises, capable of causing severe burns several kilometers from the detonation point.^18^ The development of “burns” caused by ionizing radiation and radioactive fall-out are common later sequelae that diminish in frequency and severity as distance from the detonation increases.

## WHERE DO BURNS FIT IN TERMS OF THE CLASSIFICATION OF DAMAGE ENSUING FROM BLASTS?

In the case of conventional explosive agent blasts, the classification of injuries generated consists of 4 groups:

1. Primary (barotrauma) caused by exposure to over- or underpressurization relative to atmospheric pressure. Injuries occur primarily to gas-containing hollow organs at air-liquid interfaces. Injuries to lungs, tympanic membranes, and bowel predominate with tympanic membrane rupture, the commonest primary injury.

2. Secondary (fragmentation) consists mainly of penetrating and blunt trauma caused by objects energized by the blast wind such as glass fragments and metallic particles. A proportion of these may have had their origin from the explosive casing. These injuries are the most common reason for hospital treatment after a blast.

3. Tertiary (displacement) consists mainly of blunt injury as a result of violent displacement of the patient and large objects by the blast wave. Contusions, fractures, dislocations, and lacerations frequently result.

4. **Quaternary (miscellaneous). These injuries are not caused by the blast itself but as a result of building collapse (crush injuries), burns from fires started by the explosion, exposure to toxins and poisons (respiratory distress and asphyxiation), radioactive particles in a “dirty bomb” (radiation burns/sickness), and infectious disease agents distributed by the explosion (eg, anthrax spores).**

However, thermobaric agents, which are a subcategory of volumetric weapons (which also includes fuel-air explosives), are designed to create exaggerated pressure and thermal effects. The injury classification for these agents has not been as formally defined as for conventional high explosives with most authors concentrating on barotrauma effects because of the exaggerated overpressure that thermobaric agents generate.^12^,^15^,^16^ However, even the name suggests that their primary injury mechanisms are heat and pressure with secondary injury mechanisms caused by flying particulate matter created by the interaction of the blast with nearby structures (eg, flying masonry, glass shards, and metallic debris) plus asphyxiation by the generation of toxic gases and smoke.

## HOW BURNS OCCUR IN THESE INCIDENTS

a. Primary: Related to the explosive device itself and the amount of heat/flame/fireball generated by the explosion

b. Secondary: Related to later phenomena

1. Flame burns caused by clothes catching fire

2. Flame burns sustained from environment fires caused by the blast (ie, furniture) while attempting to self-extricate/escape

3. Contact burns sustained by being trapped by hot materials/surfaces or touching hot surfaces while attempting to self-extricate/escape

4. Electrical burns from power lines brought down/exposed

5. Radiation burns if “dirty” bomb or nuclear device

## WHY ARE BURNS DIFFERENT FROM OTHER FORMS OF TRAUMA?

Major burn injuries are the most complex trauma cases to manage.^19^ The pathophysiological effects of burn injuries evolve with time. The likelihood of surviving these injuries is not merely a function of the burn injury itself, compounded by the presence of coexisting medical problems and concomitant nonburn trauma but is a multifactorial equation that includes the availability and adequacy of burn care resources and, in some situations, fiscal constraints.

There is a global paucity of specialists involved in tertiary burn care and it seems this shortage extends to those interested in pursuing a career in this field. Most burn units are equipped to manage the day-to-day burn workload generated by their catchment area and little reserve exists to cope with much in the way of a “disaster surge.” The shortage includes other burn care personnel such as specialist burn-trained nurses, allied health staff, psychologists, social workers, and counselors. The deficiency extends also to appropriate hospital beds on both burn units and intensive care units (ICUs). Because of budgetary constraints, all the other resources needed for the timely and appropriate management of burn victims, such as emergency operating theatres and staff and pathology and radiology services, are also designed to cope with the “everyday” workload. All of these factors have major implications for the scheduling of the multiple parallel and serial episodes of surgery that is necessary following burn disasters. The inability of acute burn services to cope with major surge is exceeded only by that displayed by post–acute burn rehabilitation units.

It is to be hoped that in first-world nations, there are no fiscal considerations affecting provision of care. Prolonged ICU and burn unit bed stays are extremely expensive, as is the increasing use of bioengineered skin substitutes. The acute care of a single major burn (>50% TBSA) often costs more than A$0.5M. Disaster surges deplete blood stores and cadaver skin bank stock. Repeated episodes of surgery, followed by rehabilitation, medium-term and long-term reconstructive surgery, may have visible costs but these are negligible when compared with temporary or lifelong loss of productivity, health insurance claims, criminal injury payments, disability payments, etc.

## DISASTER PLANNING

Different considerations apply to every small country, and each region within larger nations, in developing a major burn incident response plan. These include population density; geographical factors such as the geometric area covered and the variations in terrain within that area; road, rail, waterway, and air routes for access and retrieval by emergency services; seasonal weather variations; capacity and adequacy of receiving (primary) and definitive (tertiary) hospital services; political situation and the potential involvement of military services; and, most importantly, funding. Within this framework, each country or state should be aware of the resources available and design the optimal response. Any plan should enable the most appropriate response, given the available resources to ensure the best possible outcome.

It becomes obvious when reading accounts of disaster plan development in the United States that the “gold standard” of care possible with almost unlimited facilities and funding, supported at state and federal government levels and coordinated by the military, is almost impossible to emulate anywhere else in the world. Disaster contingency plans of any kind constitute a form of insurance. There is, happily, no guarantee that these plans will ever require activation. The downside of this situation is that attracting the time and input of the specialists who need to be involved and the administration that needs to provide the infrastructure, coordination, and the requisite funding can be difficult. The issue becomes a risk management strategy in planning and funding.

In the United States, there are 2367 burn beds available in 225 American Burn Association–recognized burn centers and 145 burn beds in other hospitals capable of looking after burns casualties, servicing a population of approximately 295 million (1 bed per 117,436 people). In South Australia, for example, with a population of 1.52 million (of which 73.1% [1.11 million] live in metropolitan Adelaide), there are a total of 8 adult burn beds (1 bed per 189,875 people). These beds also serve the Northern Territory (197,000 people) as well as areas of New South Wales (Broken Hill, 23,000 people) and Victoria (Mildura, 44,000 people), decreasing the bed per population ratio to 1:222,875.

In the United States, with the exception of Alaska, no area is more than 300 km from an accredited burn center, compared with South Australia, where some populated industrial areas such as Moomba and Roxby Downs are 800 km and 750 km from the nearest burn center (Adelaide). Alice Springs with a population of 25,000 is 1275 km from Darwin (which manages simple burns up to 20% TBSA) and 1325 km from Adelaide. The total catchment area for the adult burns unit at the Royal Adelaide Hospital (RAH) is 2.4 million square kilometers. The number of retrieval aircraft for Australia is also on a small scale compared with that for the United States and at the moment no contingency plan allows for routine deployment or availability of military aircraft. Even trying to adapt elements of the UK burn disaster plan for use in Australia is pointless. Population densities and the number of burn units and facilities in the United Kingdom are completely different from the Australian situation, coupled with the United Kingdom having a much smaller land area negating many of the problems associated with transfer.

## STRATEGIES FOR COPING

### Education

A few burn units around Australia have embarked upon the delivery of education packages to rural medical and nursing practitioners, ambulance, fire service, and police personnel. The aim of such continuing medical education is ostensibly to update such practitioners as to advances in burn care that are available at the tertiary center. Such packages also provide the opportunity to educate and/or update first-aid and primary care priorities, reminding of the need for appropriate and timely referral after assessment and resuscitation. Part of the motive for these sessions, which are time consuming to produce and costly to run, is to improve the physiological status of the patient prior to transfer to the tertiary unit and to facilitate earlier discharge of treated burn patients back to their own communities. These packages, however, allow face-to-face contact of primary practitioner and burn specialist. This facilitates subsequent referral of, or telephone discussion regarding, rural cases. On the other hand, it allows the burn specialist to view the variety of rural resources, information that may be invaluable later in establishing the primary care center as the site of trauma team and burn assessment team activity. The sessions recently held in South Australia have included a disaster component to raise awareness of the logistical difficulties posed by large numbers of burn-injured and prompt rural practitioners to hypothesize their response to a local disaster situation while informing them of the mobile resources available from the tertiary care center.

A Burn Link Nurse program has been established in South Australia to provide training for rurally based registered nurses to gain contemporary and ongoing knowledge related to the treatment of burns in the major regional center. These nurses maintain close communication and liaison with the burns unit using telemedicine, digital photography and e-mail, online education, and personal communication. This strategy was developed to facilitate discharge home of rural patients with specialized burn dressings in situ. However, further value has been foreseen since, of the 400 patients injured with burns in the Black Saturday fires, 385 were managed in their communities.

### Burns assessment teams

There has been considerable interest by several burns surgeons globally in the concept of burns assessment or burns response teams (BATs or BRTs). The role of these teams is triage, resuscitation, assessment, escharotomy, dressing, and dispersal of burn victims from the primary referral center emergency or ICU department to the most appropriate regional burns centers. The designated primary burn surgeon decides the composition and method of dispatching the BAT. The team is usually led by a senior and experienced burns surgeon, involves an experienced burns nurse with more than 2-year burns experience (preferably EMSB trained) and a burns-experienced registrar who is not on call. In South Australia, this role could also be filled by a plastic surgeon not required at the team's base hospital. Generally, the members of the team wear identifiable tabards and carry burn assessment packs, documentation, and protocols for use by the primary emergency department (ED), health facility, or site.

The issue in all centers investigating the possibility of BAT deployment is how the team would be transported to the receiving primary ED. In the United Kingdom, the teams are dispersed via hospital transport cars provided by ambulance control. However, in South Australia, the potentially enormous distances between the local ED and the RAH make this idea largely unworkable. One solution, in the event of fire disaster where there are likely to be many seriously injured patients requiring ICU retrieval, is for the BAT to travel with the MedStar Retrieval Service. The BAT would likely remain at the receiving primary center until all casualties requiring transfer to an appropriate tertiary center (not necessarily the RAH) had been transported out. Only then would the team return to the RAH so that the inconvenience of the team taking up space in the retrieval aircraft would be “one way.”

The first role of the BAT is to assess the facilities for burns patients at the receiving hospital and to supplement equipment and dressings stores with their own carried stock. This is important because in any disaster scenario, the simplest but most essential stores are rapidly depleted and primary centers have limited materials for dressing a *single* major burn–injured patient, let alone *multiple* burn–injured patients. Next is facilitating the processes of triage, assessment, resuscitation, and dressing to make the patient fit for transfer. The performance of specialist emergency surgical procedures, such as escharotomy, may be necessary if prolonged time to transfer is likely. The presence of the BAT should allow filtering of patients in a coordinated, prioritized fashion; hopefully, in such a way that the tertiary center does not become decompensated by a sudden influx of hastily transferred and inadequately triaged/resuscitated patients. It also allows burns assessment of patients who will subsequently be transferred to the ICU enabling coordinated care on arrival. This system should reduce disaster traffic through the tertiary center ED enabling the staff to concentrate on disaster victims with recognized other, nonairway, nonburn injuries while maintaining the routine trauma activity. Finally, the decision that burn resources in other states (and even other countries) will be needed should be made early and appropriate communication established to facilitate dispersal of patients from the disaster area to other, more distant, tertiary burn centers following stabilization. Once patients arrive at the nearest tertiary center and overwhelm its resources, transfer on to other tertiary units is more difficult and unlikely to occur in the early stage. The prime situation for the SA BAT dispersal is likely to be rural industrial, transport, or bushfire disaster, where the time to retrieval from the disaster site to tertiary services will be prolonged and transport resources will be scant. It is unlikely that the BAT will be deployed in a burn disaster occurring in the metropolitan Adelaide area (where the greatest population density resides) because patients will arrive at the tertiary centers early, whether by ambulance, car, or walking.

### Resource-based disaster planning

When a burn disaster occurs in a metropolitan area, there is likely to be no role for the BAT. Coping with an immediate surge of patients through the EDs and the early surge for burn services and intensive care services mandates a thorough knowledge of what resources (medical and nursing personnel, special beds, theatres and anaesthetists, etc) are available immediately, and how subsequently recruited resources can be organized to provide ongoing “shift” care. Close collaboration with representatives of major dressing manufacturers, cadaver skin banks, etc, is essential if critical delays in product arrival are to be avoided. Resource-based planning is something of a rarity and the authors of disaster plans tend to concentrate on the “administrative” aspects of the response. This stems largely from the desires of their political masters, the source of funding for plan development. However, there seems little point in having the “chiefs” organized when there are no “Indians.” Such a resource-based plan is under development in South Australia. To illustrate what this entails, I will provide one component of the plan as an example. Since the most crushing deficiency involves burn surgeons, a database has been established following canvassing of all plastic and reconstructive surgeons in the metropolitan Adelaide area. A 4-category designation has been proposed and surgeons have been encouraged to suggest to which category they belong. The categories have been designated primary, secondary, tertiary, and quaternary. A primary burn surgeon is involved in burn care, including major burn care (>50% TBSA) on a daily basis, treats over 100 burn cases per annum, and is familiar with local resources and interstate burn surgeons/facilities. The secondary burn surgeon is involved frequently with smaller (<25% TBSA) burn management, caring for 40 to 100 cases per annum. The tertiary surgeon has the requisite skills to be an invaluable part of a burn surgery team (skin graft harvesting/meshing, use of debriding knives, some experience with skin substitutes, etc) but does not routinely offer burn care in his/or her day-to-day practice. The quaternary surgeon will not have received any burn surgery training but will be an experienced pair of hands in the emergency situation. The role of the primary surgeon will be to plan and oversee all burn surgery and postoperative care with the individual burn surgery teams. Structured (and published^20^) surgical burn care protocols will facilitate this planning stage and laminated copies are available to be employed at all designated sites. He/or she will not perform surgery but will move from theatre to theatre providing advice and encouragement as well as liaising with primary burn surgeons at other sites and interstate resources and monitoring the supplies of surgical provisions. Each case will be treated by a burn surgery team led by a secondary burn surgeon who will perform the burn surgery for an individual case, guiding and supervising the tertiary and quaternary surgeons on the team. The database enables immediate contact with surgeons who are willing and able to help and allows them to detail their availability and their commitments. Parallel and serial episodes of surgery can thus be appropriately staffed. This surgical plan is one small example of how resources can be identified prior to an incident and marshaled once the incident occurs. A similar database for burns nurses, designating them into similar categories, has also been designed. In addition, provision has been made to recruit agencies that do not routinely offer acute burn care and arrangements are tacitly in place for any “walking wounded” to be taken to a Royal District Nursing Service (RDNS) center for assessment and dressing, reducing the burden on tertiary services. Education to make this possible is being provided. The ad hoc, hurried, and often-frantic identification of such resources during an incident is simply inadequate and frankly dangerous.
